# Prevention of bleomycin-induced pulmonary fibrosis by a RANKL peptide in mice

**DOI:** 10.1038/s41598-022-16843-7

**Published:** 2022-07-21

**Authors:** Nan Ju, Hiroki Hayashi, Munehisa Shimamura, Satoshi Baba, Shota Yoshida, Ryuichi Morishita, Hiromi Rakugi, Hironori Nakagami

**Affiliations:** 1 Department of Health Development and Medicine, Osaka University Graduate School of Medicine, Suita, Japan; 2 Department of Geriatric and General Medicine, Osaka University Graduate School of Medicine, Suita, Japan; 3 Department of Neurology, Osaka University Graduate School of Medicine, Suita, Japan; 4 Department of Clinical Gene Therapy, Osaka University Graduate School of Medicine, Suita, Japan; 5 Department of Health Development and Medicine and Department of Neurology, Osaka University Graduate School of Medicine, Centre of Medical Innovation and Translational Research (6Th Floor, Room 0612B), Osaka University, 2-2 Yamada-oka, Suita, Osaka 565-0871 Japan

**Keywords:** Chronic obstructive pulmonary disease, Autoinflammatory syndrome

## Abstract

Despite the recent therapeutic developments for the treatment of pulmonary fibrosis, its prognosis is still not well controlled, and a novel therapeutic agent is needed. Recently, the critical role of Toll-like receptors (TLRs) in the pathophysiology of pulmonary fibrosis has been reported; however, the effects of multiple TLR signaling inhibition are still unknown. Here, we examined how the inhibition of multiple TLRs affects pulmonary fibrosis using a novel synthetic receptor activator of nuclear factor κB ligand (RANKL) partial peptide, MHP1-AcN, which could suppress TLR2, 3, 4, 7, and 9 signaling through CD14 and RANK. When MHP1-AcN was administered in the bleomycin-induced lung fibrosis model, reduced collagen deposition was observed, with suppressed fibrosis-related gene expression including *Col1a1*, *Col1a2*, *Acta2*, *Tgfb1* and *Tgfbr2*. MHP1-AcN also decreased proinflammatory M1 and profibrotic M2 macrophage marker expression. Furthermore, MHP1-AcN treatment inhibited transforming growth factor (TGF-β)-induced Smad2/3 phosphorylation and myofibroblast differentiation in human fetal lung fibroblast (MRC-5) cells. This effect was associated with decreased TGF-β receptor levels and the upregulated *Bmp7* and *Smad7* expression. These findings suggest that MHP1-AcN protects mice against bleomycin-induced pulmonary fibrosis. MHP1-AcN might provide a novel therapeutic strategy for the pulmonary fibrosis.

## Introduction

Pulmonary fibrosis (PF) is a progressive disease featuring structural destruction, fibroblast proliferation and excessive accumulation of collagen, resulting from varying chronic inflammatory disorders in lung^1,2^. Although the type I and II procollagen production inhibitor, pirfenidone, and the tyrosine kinase inhibitor, nintedanib are in the market, the prognosis of PF is still poor, and development of novel therapy is needed. Recently, the involvement of Toll-like receptors (TLRs) in the pathophysiology of lung fibrosis has been demonstrated as a key factor in the disease. TLRs recognize damage-associated molecular pattern (DAMP) released from injured epithelial cells, resulting in production of proinflammatory cytokines and profibrotic mediators that promote lung fibrosis^3^. Some TLR inhibitors were reported to be effective although they were in preclinical studies. For instance, TAK242, a specific inhibitor of TLR4 signaling, suppresses pulmonary fibrosis in a preclinical fibrosis model induced by subcutaneous bleomycin administration^4^. TLR2 neutralizing antibody improves survival and protects mice from the development of bleomycin-induced lung fibrosis^5^. TLR9 is highly expressed in patients with rapidly progressive idiopathic pulmonary fibrosis (IPF) and its agonist exacerbates fibrosis in a humanized SCID mouse model of IPF^6^. Furthermore, TLR3, 4 and 9 pathways have been demonstrated to regulate transforming growth factor beta (TGF-β) expression, which is the master regulator of fibrosis^7^. However, other studies reported opposite results. For example, targeting TLR4 by genetic ablation or neutralizing TLR4 antibody shows exacerbation of lung fibrosis^8^. TLR3 has been reported to be anti-fibrotic as a synthetic TLR3 ligand mitigates profibrotic responses^9^. Although the benefit of TLR signaling for PF is still controversial, the merit of broad inhibition of TLR signaling has not been reported yet.

We previously showed that receptor activator of nuclear factor κB ligand (RANKL)/RANK signaling inhibits TLR4 signaling in activated microglia in ischemic stroke^10^ and another showed similar effects on TLR4, 5, and 9 signaling in bone marrow-derived macrophages^11^. Based on these findings, we developed a partial RANKL peptide, MHP1-AcN, including DE (residues 245–251, bridging β-strands D and E) and a part of EF loop (residues 261–269, bridging β-strands E and F) in RANKL, but not AA’ (residues 170–193) and CD loops (residues 224–233) which are responsible for osteoclastogenesis. This peptide exerts inhibitory effects on TLR 2, 3, 4, 7/8, and 9-mediated inflammatory cytokine production without inducing osteoclast activation^12,13^. MHP1-AcN binds to CD14 and RANK, and its anti-inflammatory effect is dependent on both molecule^12,13^. Compared to other small molecules targeting CD14, such as IAXO-101 and VB-201, whose reactions are limited to TLR2 and TLR4 signaling^14,15^, the broad inhibitory effect of this peptide on TLRs is characteristic. Taking advantage of this characteristic, we have examined its therapeutic effects in the following animal models: ischemic stroke, which is associated with TLR2 and TLR4^16^; psoriasis associated with TLR7 and TLR8^17^, and LPS-induced acute lung injury associated with TLR4 and TLR9^13^. Based on this background, we hypothesized that MHP1-AcN is effective for the prevention of bleomycin-induced pulmonary fibrosis. We examined the collagen deposition and fibrosis-related gene expression in lungs of mice. We also checked the effect of MHP1-AcN on TGF-β and TLR signaling in human fetal lung fibroblast (MRC-5) cells.

## Results

### MHP1-AcN protected mice against bleomycin-induced lung fibrosis

**Figure 1:**
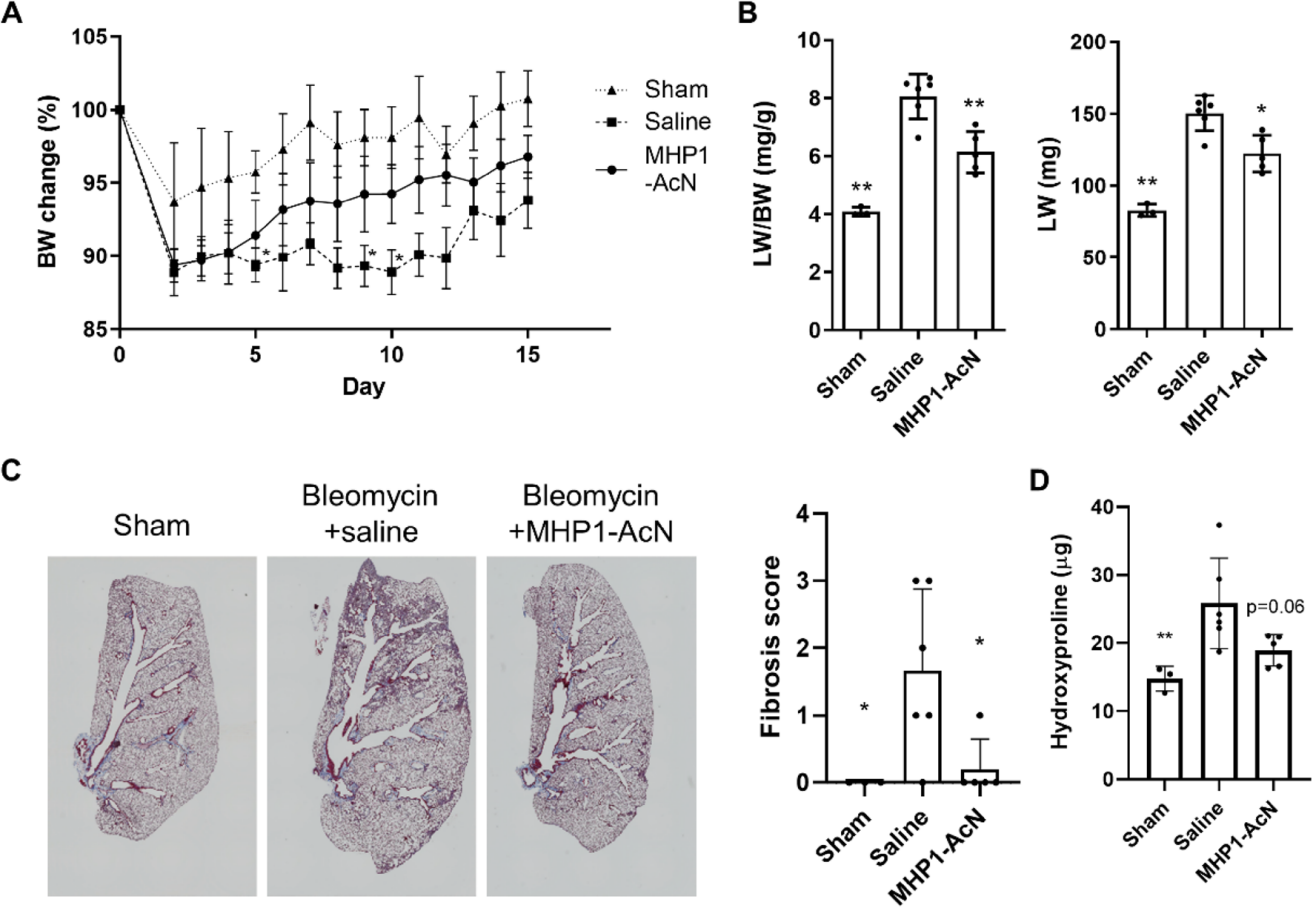
MHP1-AcN prevented the progression of bleomycin-induced lung fibrosis. (**A**) Change of body weight after intrathecal administration of bleomycin. MHP1-AcN (800 µg/day) was started immediately before intrathecal injection and continued for 14 days. Sham-operated mice received saline instead of bleomycin. Recovery of body weight was delayed in saline-treated mice. *p < 0.05 vs sham-operated mice. (**B**) Lung weight (LW) to body weight (BW) ratio (LW/BW) (left panel) and LW (right panel). MHP1-AcN inhibited increases in LW/BW and LW. *p < 0.05, **p < 0.01 vs Saline-treated mice. (**C**) Representative images of Masson’s trichrome stain (left panel) and semi-quantification of fibrosis score (right panel). MHP1-AcN inhibited fibrosis. *p < 0.05 vs Saline-treated mice. (**D**) Hydroxyproline content in lungs of mice. **p < 0.01 vs Saline-treated mice. n = 3 in sham-operated group, n = 6 in saline-treated group, n = 5 in MHP1-AcN-treated group.

**Figure 2:**
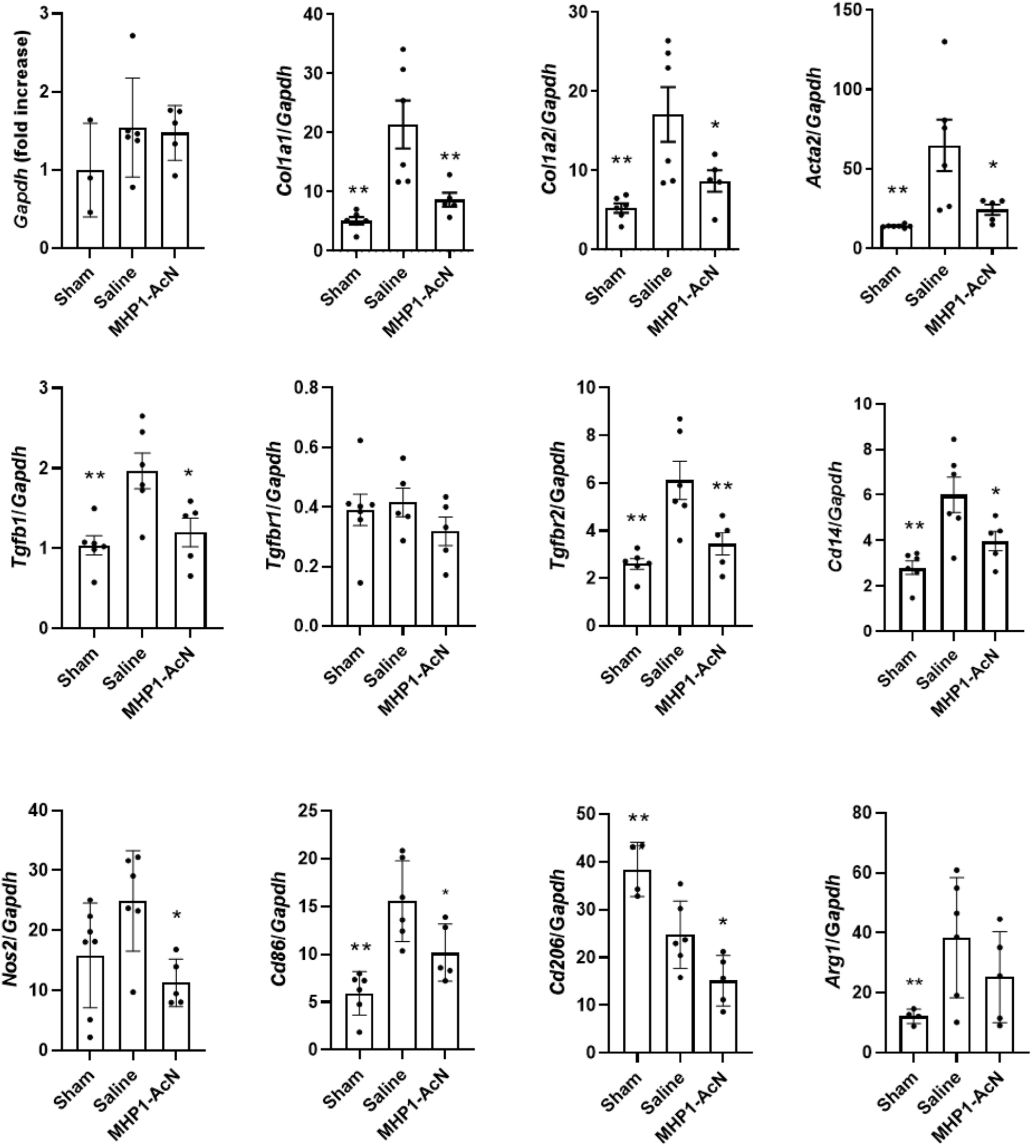
MHP1-AcN prevented the progression of bleomycin-induced lung fibrosis. Expression of fibrosis (*Col1a1* and *Col1a2*), myofibroblast (*Acta2* (αSMA)), TGF-β signaling (*Tgfb1*, *Tgfbr1*, *Tgfbr2*), M1 marker (*Nos2* (iNOS), *Cd86*), M2 marker (*Cd206*, *Arg1*)-related genes, and *Cd14* on day 14. There were no differences in *Gapdh*. *p < 0.05, **p < 0.01 vs saline-treated mice. n = 3–6 in sham-operated group, n = 6 in saline-treated group, n = 5 in MHP1-AcN-treated group.

**Figure 3:**
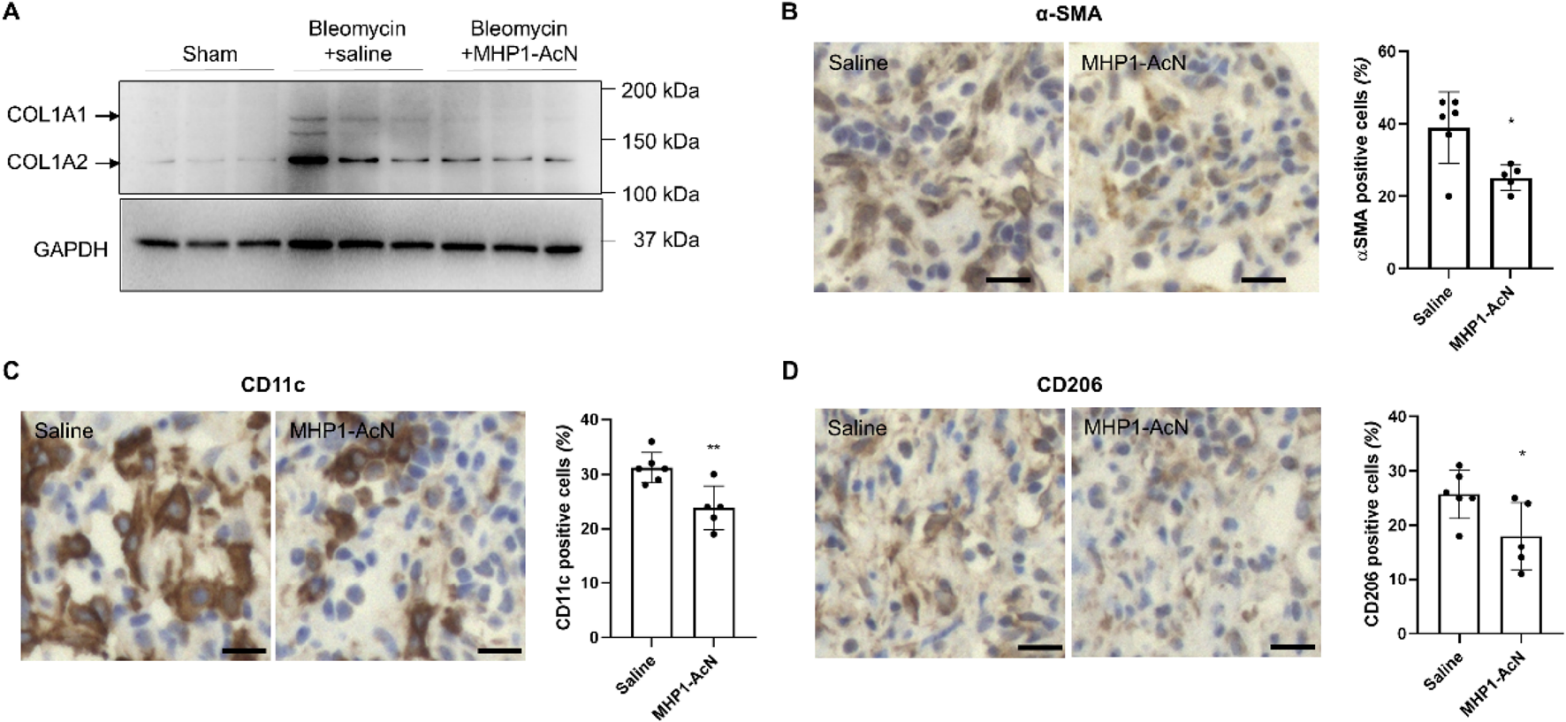
MHP1-AcN prevented the progression of bleomycin-induced lung fibrosis. (**A**) Representative western blotting images of COL1A1 and COL1A2 levels in lungs of mice. Representative images and quantification of α-SMA (**B**), CD11c (**C**) and CD206 (**D**) expression in the lungs of mice by immunohistochemistry staining. Bar = 25 µm. *p < 0.05, **p < 0.01 vs saline-treated mice.

First, we examined whether intraperitoneal injection of MHP1-AcN could prevent intrathecal bleomycin-induced lung fibrosis in mice. The body weight (BW), lung weight (LW), and the ratio of LW to BW are widely used for monitoring the health status of bleomycin-injured mice^18,19^. Compared to bleomycin-treated mice that received saline, the mice receiving MHP1-AcN showed faster recovery of BW loss (Fig. 1A), decreased loss of LW and the ratio of LW/BW on day 14 (Fig. 1B). Masson’s trichrome staining showed that bleomycin induced substantial collagen deposition in lungs of mice, which was significantly reduced by MHP1-AcN treatment (Fig. 1C). MHP1-AcN-treated mice also showed a tendency for a reduction of hydroxyproline, which is a major component of collagen (Fig. 1D). Consistent with these data, MHP1-AcN-treated mice showed suppressed expression of fibrosis-relate gene expression including *Col1a1*, *Col1a2*, *Acta2*, and *Tgfb1* compared to saline-treated mice (Fig. 2). The reduced COL1A1, COL1A2 (Fig. 3A, Supplementary Fig. 1A) and α-SMA (Fig. 3B) were also confirmed by western blotting or immunohistochemical analysis. Interestingly, bleomycin-treated mice that received saline showed an increase in *Tgfbr2* expression*,* which was inhibited by MHP1-AcN treatment, although the expression of *Tgfbr1* was not affected even in the mice receiving saline (Fig. 2). Because CD14 serves as a co-receptor for multiple TLRs^20^, we also examined CD14 expression. As MHP1-AcN was previously shown to inhibit the expression of CD14 in LPS-stimulated bone marrow-derived macrophages^13^, the upregulated CD14 expression in lung by bleomycin was inhibited by MHP1-AcN. In general, CD14 is expressed in macrophages, whose dysfunction plays a critical role in promoting lung fibrosis^21^. Sustained activation of proinflammatory M1 macrophages by acute lung injury initiates fibrotic responses in the lung, while the infiltration of profibrotic M2 macrophages in the lung functions as a critical regulator in the aberrant development of lung fibrosis^22^. From the viewpoints, we examined the effects of MHP1-AcN on macrophages in fibrotic lung^23^. The bleomycin-treated mice that received MHP1-AcN showed suppressed M1 markers (*Nos2* and *Cd86*) and M2 markers (*Cd206* and *Arg1*) expression compared to saline-treated mice; however, *Arg1* did not reach significant difference (Fig. 2). Immunohistochemical analysis also showed reduced percentage of CD11c-positive M1 (Fig. 3C) and CD206-positive M2 macrophages (Fig. 3D). Collectively, these results indicate that MHP1-AcN protected mice against bleomycin-induced lung fibrosis.

### MHP1-AcN inhibited TGF-β1-induced Smad2/3 phosphorylation and myofibroblast differentiation in fibroblasts

**Figure 4:**
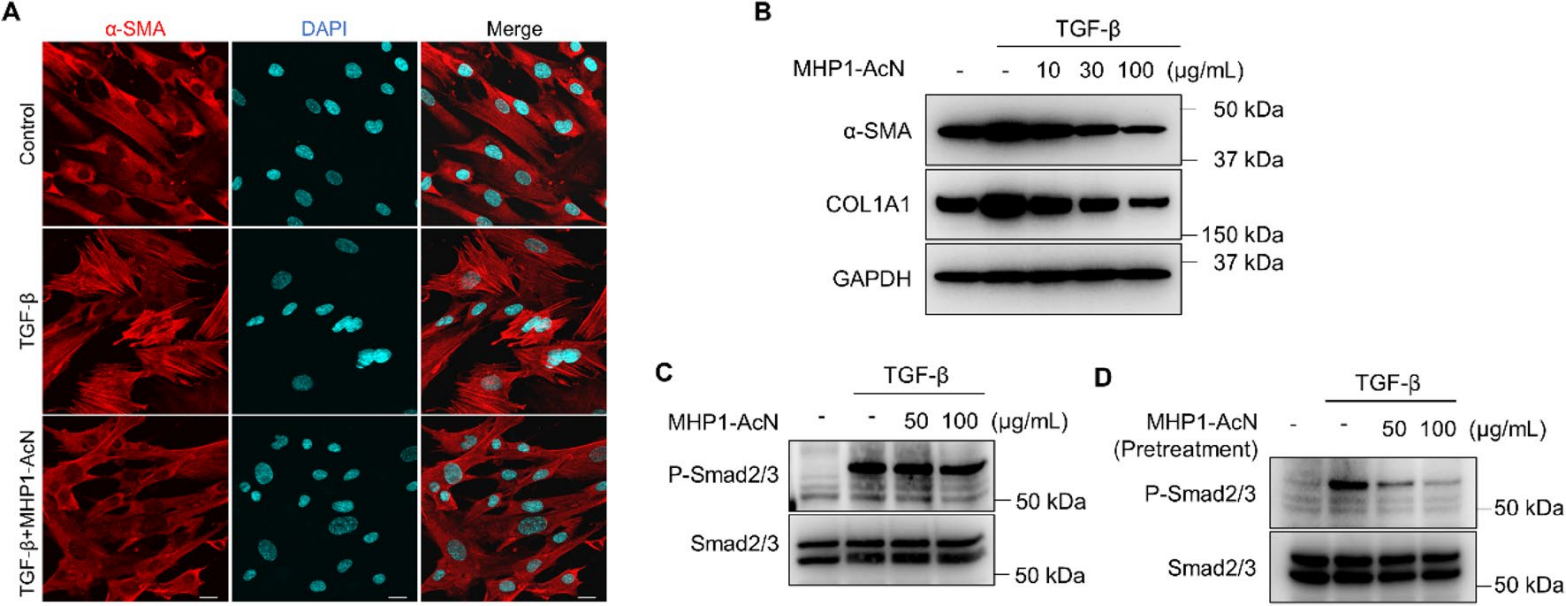
MHP1-AcN inhibited TGF-β1-induced myofibroblast differentiation and Smad2/3 phosphorylation in fibroblasts. (**A**) MRC-5 cells treated with TGF-β1 (5 ng/mL) with or without MHP1-AcN (50 µg/mL) for 48 h were stained with anti-α-SMA and DAPI and viewed by confocal microscopy. Bar = 20 µm. (**B**) MRC-5 cells were treated with TGF-β1 (5 ng/mL) with or without MHP1-AcN (10, 30 or 100 µg/mL) for 48 h, the α-SMA and COL1A1 levels were analyzed by western blotting. (**C**) MRC-5 cells were simultaneously treated with TGF-β1 (2 ng/mL) and MHP1-AcN (50 or 100 µg/mL) for 30 min, the Smad2/3 phosphorylation was evaluated by western blotting. (**D**) MRC-5 cells were pretreated with MHP1-AcN (50 or 100 µg/mL) for 24 h, then incubated with TGF-β1 (2 ng/mL) for 30 min. Smad2/3 phosphorylation was evaluated by western blotting. Experiments were repeated at least twice.

Because a marker for myofibroblast differentiation, α-SMA, was suppressed in bleomycin-treated mice that received MHP1-AcN, we further examined whether MHP1-AcN effects on TGF-β1-induced myofibroblast differentiation in the fibroblast cell line, MRC-5 cells. Treatment of MRC-5 cells with TGF-β1 resulted in cellular hypertrophy and stress fibers, whereas this effect was inhibited by pretreatment with MHP1-AcN (Fig. 4A). This effect was also confirmed by western blotting analysis, which showed decreased levels of α-SMA and COL1A1 in MHP1-AcN-treated cells (Fig. 4B, Supplementary Fig. 1B).

TGF-β/Smad signaling is a major pathway which modulates myofibroblast differentiation upon TGF-β stimulation^23^, so we next evaluated whether MHP1-AcN affects TGF-β/Smad signaling in MRC-5 cells. Simultaneous treatment of MRC-5 cells with TGF-β1 and MHP1-AcN did not affect TGF-β-induced Smad2/3 phosphorylation (Fig. 4C, Supplementary Fig. 1C). However, pretreatment of MRC-5 cells with MHP1-AcN significantly inhibited Smad2/3 phosphorylation in response to TGF-β1 (Fig. 4D, Supplementary Fig. 1D). Collectively, these results suggest that pretreatment of MHP1-AcN could inhibit TGF-β1-induced myofibroblast differentiation and Smad2/3 phosphorylation in fibroblasts.

**Figure 5:**
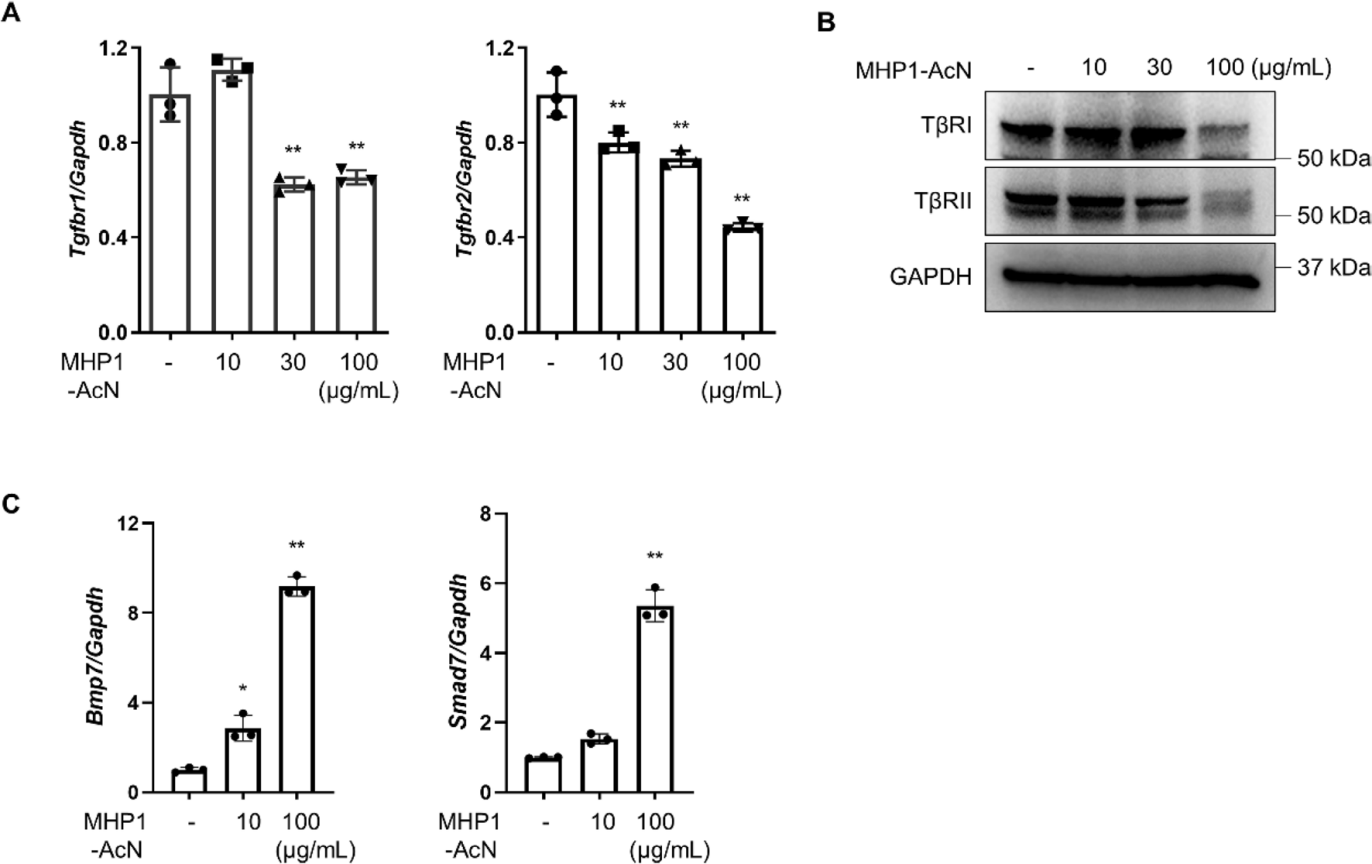
MHP1-AcN decreased TGF-β receptor expression via upregulating *Bmp7* and *Smad7* expression in fibroblasts. (**A**) qPCR results of *Tgfbr1* and *Tgfbr2* expression in MRC-5 cells after incubation with MHP1-AcN (10, 30 or 100 µg/mL) for 24 h. **p < 0.01 vs Control. *n* = 3 in each group. (**B**) Western blot analysis of TβRI and TβRII levels in MRC-5 cells after incubation with MHP1-AcN (10, 30 or 100 µg/mL) for 24 h. The experiments were repeated at least twice. (**C**) qPCR results of *Bmp7* and *Smad7* expression in MRC-5 cells after incubation with MHP1-AcN (10 or 100 µg/mL) for 24 h. *p < 0.05, **p < 0.01 vs Control. *n* = 3 in each group.

Next, we reasoned how a RANKL peptide could affect TGF-β/Smad signaling. Because the expression of *Tgfbr2* mRNA was reduced in the mice-treated with MHP1-AcN, we examined whether MHP1-AcN had influence on its expression in MRC-5 cells. When MRC-5 cells were treated with MHP1-AcN for 24 h, TβRI as well as TβRII expression was suppressed at both mRNA level (Fig. 5A) and protein level (Fig. 5B, Supplementary Fig. 1E). Since BMP7 was reported to inhibit TGF-β signaling by suppressing Smad2/3 phosphorylation and/or promoting TGF-β receptor degradation through Smad7^24–27^ we next checked the expression level of *Bmp7* and *Smad7* mRNA. As expected, MHP1-AcN treatment led to a significant increase in *Bmp7* and *Smad7* in MRC-5 cells (Fig. 5C). These data indicate that the inhibitory effect of MHP1-AcN on TGF-β signaling is, at least in part, mediated through upregulating BMP7 and Smad7, which might promote the degradation of TGF-β receptors.

### MHP1-AcN inhibited TLR9-induced IL6 secretion in fibroblasts

In addition to the stimulation of TGFβ signaling, chronic TLR9 stimulation in human lung fibroblasts was reported to cause α-SMA-positive myofibroblast transformation, with the secretion of inflammatory cytokines^28^. To clarify whether MHP1-AcN affects the TLR signaling in fibroblasts, we checked the IL-6 levels in MRC-5 cells after TLR9 (*E. coli* DNA) or TLR4 (LPS) stimulation (Supplementary Fig. 2). Unexpectedly, LPS did not affect the secretion of IL-6, whereas *E. coli* DNA-induced IL-6 was inhibited by MHP1-AcN. Thus, the inhibitory effect of MHP1-AcN on TLR9 signaling in fibroblasts might also contribute to the prevention of bleomycin-induced lung fibrosis.

### MHP1-AcN protected mice against bleomycin-induced skin fibrosis

**Figure 6:**
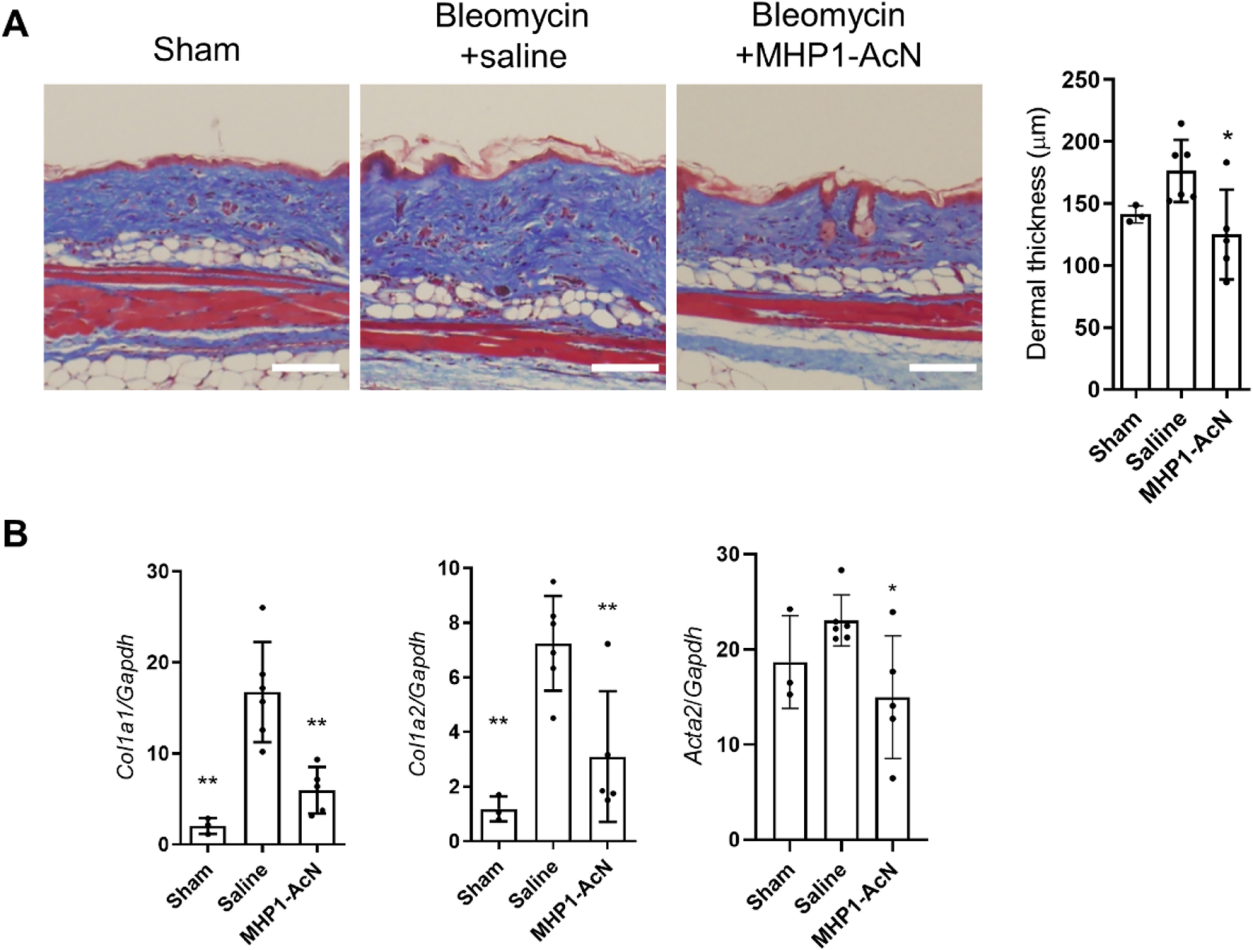
MHP1-AcN prevented the progression of bleomycin-induced skin fibrosis. (**A**) Typical images of Masson’s trichrome staining (left panel) and quantification of dermal thickness (right panel) on day 28. Bar = 100 µm. (**B**) Expression of *Col1a1*, *Col1a2*, and *Acta2* on day 28. Fibrosis was inhibited in both histology and mRNA expression in MHP1-AcN-treated mice. *p < 0.05, p < 0.01 vs saline-treated mice. n = 3 in sham-operated group, n = 6 in saline-treated group, n = 5 in MHP1-AcN-treated group.

Finally, we examined bleomycin-induced skin fibrosis to check whether the effectiveness of MHP1-AcN was limited to the lung. After injection of bleomycin for 14 days, MHP1-AcN was intraperitoneally injected daily for 14 days. Masson trichrome staining showed that bleomycin-induced dermal thickness was inhibited in MHP1-AcN-treated mice (Fig. 6A). Similar to the results in lung fibrosis, expression of *Col1a1*, *Col1a2*, and *Acta2* was inhibited in MHP1-AcN-treated mice (Fig. 6B). These results indicate that the effectiveness of MHP1-AcN is not limited to lung fibrosis.

## Discussion

In the present study, we verified the preventative effects of MHP1-AcN on bleomycin-induced pulmonary fibrosis as assessed by reduced collagen deposition and reduced number of M1 and M2 macrophages in the lungs. MHP1-AcN also suppressed TGF-β and TLR9 signaling in MRC-5 cells and bleomycin-induced skin fibrosis.

Inhibition of myofibroblast differentiation is a practical strategy to treat fibrosis-related diseases because myofibroblasts secret various cytokines^29^. Although the effectiveness of multiple inhibition of TLR signaling for myofibroblast differentiation has not been reported, TLR2, TLR4, and TLR9 have been reported to promote myofibroblast differentiation^30–32^. In skin fibroblasts, LPS-triggered TLR4 activation alone did not activate TGF-β/Smad signaling but promoted differentiation into myofibroblasts with enhanced TGF-β responses in the presence of TGF-β^33^. However, fibronectin extra domain A or tenascin-C-triggered TLR4 activation induced fibrotic responses in human skin fibroblast, which were abrogated by TLR4 inhibitor^31^. Considering that LPS did not affect IL-6 production in MRC-5 cells in our study, TLR4 activation, which could promote fibrotic responses, might be ligand-specific and cell type-specific. Despite no involvement of TLR4, the inhibition of IL-6 production was shown in TLR9-stimulated MRC-5 cells. This indicated that the protective effects of MHP1-AcN might also be attributed to the direct inhibition of TLR9 signaling in fibroblasts.

In general, TGF-β1 is produced by a wide variety of cell types, including endothelial cells, fibroblasts, myofibroblasts, alveolar macrophages, neutrophils, and activated alveolar epithelial cells^34^. In the present study, the cells responsible for the decreased *Tgfb1* expression were not clarified, but the reduction of *Cd206* and *Arg1* expression indicated a decrease in M2 macrophages, which reportedly produced profibrotic mediators, such as TGF-β and PDGF^22^, as one of the causes. TGF-β signals are transduced by TGF-β receptors to modulate the profibrotic effects including myofibroblast differentiation, fibroblast proliferation and extracellular matrix accumulation^35^. In the present study, MHP1-AcN decreased TβRI and TβRII expression in fibroblasts, but in bleomycin-induced lung fibrosis model, MHP1-AcN treatment inhibited the increase in *Tgfbr2* without significant change in *Tgfbr1* on day 14 after bleomycin exposure. The reasons for these discrepancies are not clear, but the time course of *Tgfbr1* is likely to contribute, as TβRI was reported to be significantly upregulated on day 7 post bleomycin^36^. The increased expression of *Bmp7* and *Smad7* indicated that the degradation of TGF-β receptors might be one of the mechanisms of reduced expression of TGF-β receptors, but how MHP1-AcN affects these molecules and the link between TLR signaling and TGF-β signaling needs further investigation. In previous studies, BMP7 was reported to have beneficial effects in many fibrotic models including renal fibrosis and cardiac fibrosis^37,38^, but BMP7 administration was insufficient to protect mice against bleomycin-induced pulmonary or skin fibrosis^39^. Therefore, the combination of anti-TLR signaling and upregulation of *Bmp7* by MHP1-AcN might be ideal for the prevention of bleomycin-induced pulmonary fibrosis.

The limitation of this study is that we could not exclude the possibility that the anti-inflammatory effects of MHP1-AcN in bleomycin-induced lung fibrosis model might be the cause of anti-fibrotic effects because we administered MHP1-AcN from the beginning of bleomycin. In this model, bleomycin resulted in damage to the alveolar epithelial cells and subsequent inflammatory responses during the first 7 days, and the transition from inflammation to fibrotic processes at 7–14 days post bleomycin exposure^40^. MHP1-AcN might have exerted its anti-fibrotic effects only through its anti-TLR effects for the initial injury and inflammatory responses. However, we believe that the effects of MHP1-AcN was dependent on anti-fibrosis as well as anti-inflammatory effects because MHP1-AcN suppressed TGF-β/Smad signaling and TLR9 signaling in MRC-5 cells. Another limitation of this study is examining the effect of MHP1-AcN only in a bleomycin model. Although the bleomycin-induced lung fibrosis model has been extensively used to evaluate potential therapies^41^, a single animal model does not recapitulate the spectrum of human pulmonary fibrosis. For clinical applications, further study is necessary in another animal model, such as silica-induced lung fibrosis model. Another limitation is the limited information on the action mechanism of MHP1-AcN for bleomycin-induced skin fibrosis. Although we speculate that MHP1-AcN suppresses skin fibrosis with a similar mechanism to lung fibrosis, it is necessary to clarify how MHP1-AcN prevents skin fibrosis for clinical applications.

Taken together, this study demonstrated the protective effects of MHP1-AcN in bleomycin-induced lung fibrosis model. The mechanism of this effect involved inhibition of TLR signaling and, at least in part, inhibition of TGF-β signaling via upregulation of BMP7 and Smad7 in fibroblasts.

## Materials and methods

### Peptide synthesis

Synthetic MHP1-AcN (Ac-LMVYVVKTSIKIPSSHNLMKGGSTKNWSGN-NH2) was purchased from ILS, Inc. (Ibaragi, Tsukuba, Japan), dissolved in double-distilled water (H_2_O) to make a 2 mg/mL solution, and stored at 4 °C until use.

### Animal models of fibrosis

All experiments were approved by the Institutional Animal Care and Use Committee of Osaka University (02-017-010) and carried out in compliance with the Osaka University Guidelines, which are based on the National Institutes of Health’s Guide for the Care and Use of Laboratory Animals, and with the ARRIVE guidelines. All efforts were made to minimize suffering. C57/Bl6/J female mice were obtained from CLEA Japan, Inc.

Induction of mouse lung injury by endotracheal injection of bleomycin was performed as previously described^42^. Briefly, female mice (12–13 W) were anesthetized with a mixture of butorphanol (5.0 mg/kg), medetomidine (0.3 mg/kg), and midazolam (4.0 mg/kg). After incision of the skin in the neck, the trachea was exteriorized by blunt dissection. Bleomycin solution (0.05 U/100 µL, Nippon Kayaku, Tokyo, Japan) was slowly instilled into the lumen of the trachea. The skin wound was then closed with a 5–0 absorbable suture. MHP1-AcN (40 mg/kg) was injected intraperitoneally for 14 days and sacrificed on day 14. The right lung was collected, and its weight was measured.

Skin fibrosis was induced as described previously^4^. Briefly, 8-week-old female mice received subcutaneous injections of bleomycin (10 mg/kg/day) for 10 days (5 days/week). Intraperitoneal injection of MHP1-AcN (40 mg/kg) was started on day 14 and continued to be injected daily for 14 days. The mice were sacrificed on day 28.

### Histological analysis and measurement of hydroxyproline

For Masson’s trichrome staining, the left lungs were perfused and fixed with 4% paraformaldehyde. They were embedded in paraffin, and serial 5-µm sections were stained with Masson’s trichrome stain. For immunohistochemistry, antigen retrieval was performed. The sections were blocked in 3% bovine serum albumin, followed by incubation with primary antibodies (CD11c: 97585S, Cell Signaling; CD206: ab64693, Abcam; αSMA: ab5694, abcam) overnight at 4 °C. Then the sections were incubated with Single Stain MAX PO (rabbit) Histofine (Nichirei Biosciences, Japan) followed by development with DAB (DAB substrate kit/NICHIREI). Counterstaining was performed with Meyer's hematoxylin. Images were captured using a microscope (BZ-X810, Keyence, Japan). For semi-quantification of the lung fibrosis, fibrosis was quantified using the modified Ashcroft scoring^43^. The average percentage of CD11c, CD206, and αSMA-positive cells in randomly selected 3 fields of 0.4 mm^2^ area was quantified using ImageJ (National Institutes of Health).

For the measurement of hydroxyproline, collected right lung was homogenized with saline and hydroxyproline was measured by high performance liquid chromatography (SRL, Tokyo, Japan).

### Cell culture

MRC-5 cells were obtained from the RIKEN Gene Bank (Tsukuba, Japan) and maintained in 5% carbon dioxide (CO_2_) at 37 °C in minimum essential medium α (MEM-α; Nacalai, Kyoto, Japan) supplemented with 10% fetal bovine serum (FBS; Thermo Fisher Scientific, Waltham, MA, USA) and 1% penicillin–streptomycin mixed solution (Nacalai). Cells were treated with recombinant human TGF-beta 1 protein (240-B, R&D Systems, Minneapolis, MN, USA) with or without MHP1-AcN for the indicated time. LPS (no. L4391, Escherichia coli O111:B4; Sigma-Aldrich) or *E. coli* DNA (InvivoGen) was added to the medium with MHP1-AcN at the same time. After 72 h of incubation, the medium was collected for ELISA.

### ELISA

The concentrations of IL-6 were measured using the ELISA kit: IL-6, Human IL-6 Quantikine ELISA Kit (R&D Systems). The concentration of was determined in duplicate for each sample.

### Real-time reverse transcription PCR (quantitative RT-PCR)

In the in vivo experiments, the right lung was collected 14 days after the administration of bleomycin. MRC-5 cells were plated in 12-well plastic culture dishes (1 × 10^5^ cells/well). After overnight culture, the cells were incubated with MHP1-AcN (10, 30, or 100 µg/mL) for 24 h. The mRNAs were isolated using the QIAGEN RNeasy Mini Kit (Qiagen, Germantown, MD, USA), according to the manufacturer’s recommendations. The cDNA reaction was performed using a High-Capacity cDNA Archive kit (Applied Biosystems, Foster City, CA, USA) according to the manufacturer’s instructions. The oligonucleotide primers were purchased according to the following identification: *Col1a1*: Mm00801666_g1; *Col1a2* Mm00483888_m1; *Acta2* Mm01546133_m1; *Cd14* Mm00438094_g1; *Tgfb1* Mm01178820_m1; *Tgfbr1* Mm00436964_m1; *Tgfbr*2 Mm03024091_m1; *Nos2* Mm00440502_m1; *Cd86* Mm00444540_m1; *Cd206* Mm01329359_m1; *Arg1* Mm00475988_m1; *Gapdh* Mm99999915; *Tgfbr1* Hs00610320_m1; *Tgfbr2* Hs00234253_m1; *Bmp7* Hs00233476_m1; *Smad7* Hs00998193_m1; *Gapdh* Hs02786624_g1 (Applied Biosystems). The 5’ nuclease assay PCRs were performed in a MicroAmp Optical 384-well reaction plate using a QuantStudio 6 Pro Real-Time PCR System (Applied Biosystems). The levels of the target genes were quantified by comparing the fluorescence generated by each sample with that of the serially diluted standard. In animal models, the target gene expression was normalized to the level of *Gapdh* expression in each individual sample. In in vitro experiments, the expression level of specific genes was normalized to *Gapdh* and quantified using the ΔΔCt method.

### Western blotting

Western blotting and immunostaining were performed as previously described^13^. Briefly, cells were washed twice with chilled PBS and lysed with lysis buffer (NP-40), and protease inhibitor cocktail (Roche Applied Science, Indianapolis, IN, USA). Collected lungs were disrupted and homogenized in RIPA buffer (Wako Pure Chemical Industries, Osaka, Japan) using TissueRuptor II (Qiagen, Germantown, MD, USA). Blotted membranes were incubated overnight at 4 °C with primary antibodies and washed with TBS containing 0.1% Tween-20 before incubation with HRP-conjugated secondary antibody, followed by Chemi-Lumi One L (Nacalai).

### Immunohistochemistry in cultured cells

For immunostaining, cells on glass-bottom dishes were fixed in 4% paraformaldehyde and permeabilized with 0.2% Triton X-100. The samples were blocked in 5% skim milk, followed by incubation with primary antibodies overnight at 4 °C. The corresponding secondary antibodies were labeled with AlexaFluor 546 (Thermo Fisher Scientific). Nuclear staining was performed using 4,6-diamidino-2-phenylindole (DAPI). Images were collected using a confocal microscope (FLUOVIEW FV10i; Olympus, Tokyo, Japan). Antibodies and reagents employed in western blotting and immunostaining included: anti-α SMA (A2547, Sigma-Aldrich; Merck KGaA, Darmstadt, Germany), anti-collagen I (ab34710, Abcam, MA, USA), anti-COL1A2 (ab96723, Abcam), anti-COL1A1 (E8F4L) (Cell signaling, Danvers, MA, USA), anti-phospho-smad2 (Ser465/467)/smad3 (Ser423/425) (D27F4, Cell Signaling), anti-smad2/3 (D7G7) (Cell Signaling), anti-TGF-beta RI (MAB5871, R&D Systems, Minneapolis, MN, USA), anti-TGF-beta RII (R&D Systems), and anti-GAPDH (MAB374, Sigma-Aldrich).

### Statistical analyses

Data are expressed as mean ± SD. Data were analyzed using Prism for windows version 8 (GraphPad Software, USA). Comparison between multiple groups was performed using ANOVA followed by Dunnett’s multiple comparisons test. Comparison between two groups was performed using T-test. Differences were considered significant at p < 0.05.

## Supplementary Information

Supplementary Figures.

## Data Availability

All data generated or analyzed during this study are included in this published article (and its Supplementary Information files).
