# Supplementary material for: Prevention of bleomycin-induced pulmonary fibrosis by a RANKL peptide in mice
Source: Sci Rep. 2022 Jul 21;12:12474. doi: 10.1038/s41598-022-16843-7 (PMC9304352; doi:10.1038/s41598-022-16843-7)
Supplement: Supplementary file 1 — Supplementary Figures. [file 41598_2022_16843_MOESM1_ESM.pdf]

Prevention of bleomycin-induced pulmonary fibrosis by a RANKL peptide in mice

Nan Ju[1,2], Hiroki Hayashi[1], Munehisa Shimamura[1,3*], Satoshi Baba[1,2], Shota Yoshida[1,2], Ryuichi Morishita[4], Hiromi Rakugi[2], Hironori Nakagami[1]

1. Department of Health Development and Medicine, Osaka University Graduate School of Medicine, Japan
2. Department of Geriatric and General Medicine, Osaka University Graduate School of Medicine, Japan
3. Department of Neurology, Osaka University Graduate School of Medicine, Japan
4. Department of Clinical Gene Therapy, Osaka University Graduate School of Medicine, Japan

*Corresponding author: Munehisa Shimamura

Email addresses: shimamuu@cgt.med.osaka-u.ac.jp

Department of Health Development and Medicine and Department of Neurology, Osaka University Graduate School of Medicine, Centre of Medical Innovation and Translational Research (6th floor, Room 0612B), Osaka University, 2-2 Yamada-oka, Suita, Osaka 565-0871, Japan

Tel: +81-6-6210-8359; Fax: +81-6-6210-8360

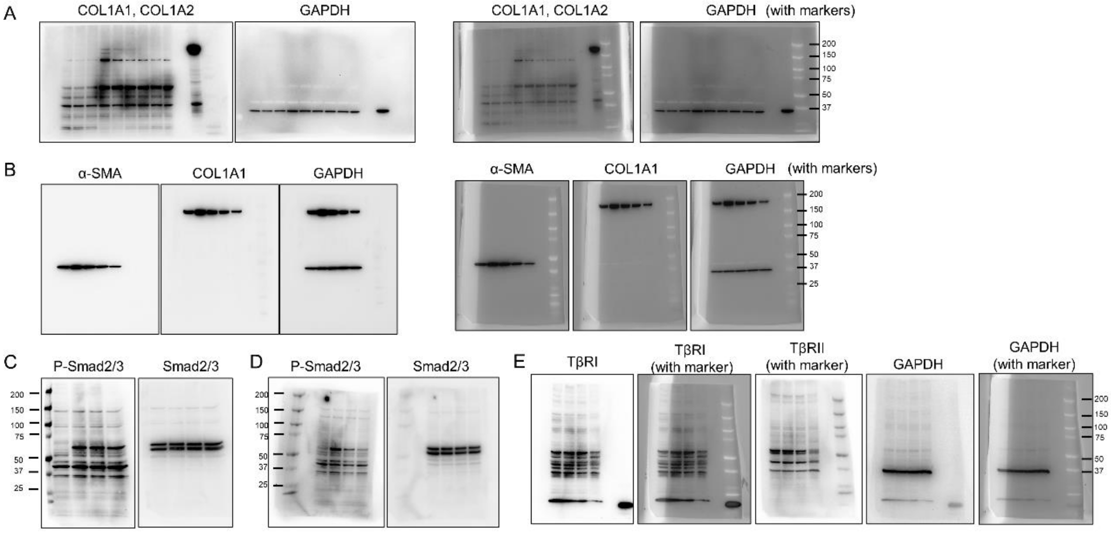

Supplementary Fig. 1. Representative Full-length blot images

A. Representative full-length blot images for Fig. 3A. **B**. Representative full-length blot images for Fig. 4B. **C**. Representative full-length blot images for Fig. 4C. **D**. Representative full-length blot images for Fig. 4D. **E**. Representative full-length blot images for Fig. 5B. Experiments were repeated at least twice.

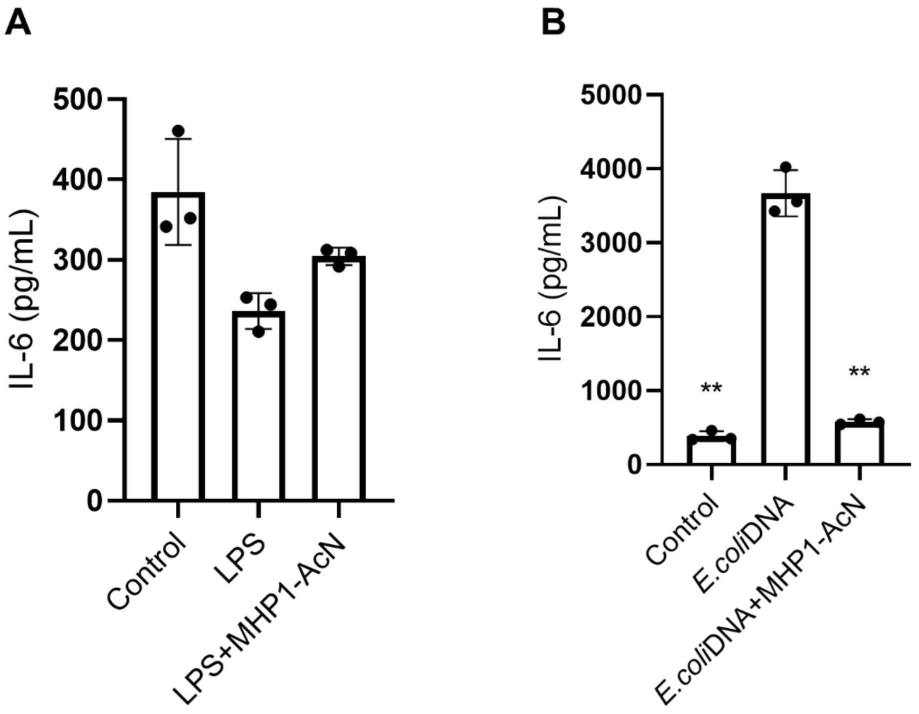

Supplementary Fig. 2. MHP1-AcN inhibited *E. coli* DNA-induced IL-6 production in MRC-5 cells.

A. MRC-5 cells were simultaneously treated with LPS (100 ng/mL) and MHP1-AcN (50 μg/mL) for 72 h, then supernatants were collected for measuring IL-6 concentration.

B. MRC-5 cells were simultaneously treated with *E. coli* DNA (10 μg/mL) and MHP1-AcN (50 μg/mL) for 72 h, then supernatants were collected for measuring IL-6 concentration. **, $p < 0.01$ vs. *E. coli* DNA. Experiments were repeated at least twice. $n = 3$ in each group.
